# Canada’s response to international travel during COVID-19 pandemic – a media analysis

**DOI:** 10.1186/s12889-021-11082-3

**Published:** 2021-05-31

**Authors:** K. Srikanth Reddy, Salima S. Mithani, Lindsay Wilson, Kumanan Wilson

**Affiliations:** 1 Bruyere Research Institute, 85 Primrose Ave, Ottawa, ON K1R 6M1 Canada; 2 WHO Collaborating Centre for Knowledge Translational and Health Technology Assessment for Health Equity, 85 Primrose Ave, Ottawa, ON K1R 6M1 Canada; 3 Clinical Epidemiology Program, Ottawa Hospital Research Institute, 1053 Carling Ave, Ottawa, ON K1Y 4E9 Canada; 4 Department of Medicine, University of Ottawa, 451 Smyth Rd, Ottawa, ON K1H 8M5 Canada

**Keywords:** Canada, COVID-19, International travel, Travel restrictions, IHR 2005

## Abstract

**Background:**

The media play a critical role in informing the public about the COVID-19 pandemic. Throughout the pandemic, international travel has been a highly contested subject at both the international and national levels. We examined Canadian media reporting on international travel restrictions during the pandemic, how these restrictions aligned with the International Health Regulations (IHR 2005), and how the narrative around international travel evolved over time.

**Methods:**

We analysed articles from Canada’s top three national newspapers by circulation – The Globe and Mail, The National Post and The Toronto Star - published between Jan 1, 2020 - May 31, 2020. Our search yielded a total of 378 articles across the three newspapers. After removing duplicates and screening the remaining articles, we included a total of 62 articles for the analysis. We conducted a qualitative media content analysis by using an inductive coding approach.

**Results:**

Three major themes were identified within the articles. These included: 1) The role of scientific and expert evidence in implementing travel restrictions; 2) Federal legislation, regulation and enforcement of international travel measures; and 3) Compliance with World Health Organization (WHO) guidelines in travel restriction policy- and decision-making. The federal government relied primarily on scientific evidence for implementing international travel restrictions and fully exercised its powers under the Quarantine Act to enforce travel regulations and comply with the IHR 2005. The government embraced a rules-based international order by following WHO recommendations on international travel, contributing to a delay in border closure and travel restrictions until mid-March.

**Conclusion:**

The media focussed significantly on international travel-related issues during the early phase of the pandemic. The dominant media narrative surrounded the need for earlier travel restrictions against international travel.

## Introduction

On January 30, 2020, the World Health Organization (WHO) declared the novel coronavirus (COVID-19) outbreak a Public Health Emergency of International Concern (PHEIC) [1] and raised the risk assessment for the COVID-19 outbreak from ‘high’ to ‘very high’ [2]. The outbreak was declared a pandemic on March 11, 2020 [3].

In order to prevent COVID-19 from spreading within their borders, many WHO member states imposed partial or complete border closures. The International Organisation for Migration reports that by March 23, 2020, 174 countries, territories, and areas around the world had coronavirus-related travel restrictions in place [4], impacting at least 7.3 billion people (i.e., 93% of the global population) [5]. Annex 1B of the 2005 revision of the International Health Regulations (IHR 2005) provides guidelines around measures that can be taken during a PHEIC, including quarantining travellers. However, these measures should not include unwarranted travel and trade restrictions that may harm the economies of countries that report health threats. As such, some scholars argue that by indiscriminately closing borders as part of their pandemic response, member states violated the IHR 2005 [6], while others posit that it was a necessary part of infection prevention measures [7, 8].

The media played a critical role in informing the public about the pandemic and shaping national policy responses to the pandemic in multiple countries [9–11]. Evidence suggests that media research studies catalyse policy action and social change for health and well-being [12]. Previous media research studies have predicted influenza disease outbreaks [13], shaped policy action regulating menthol cigarette smoking [14], and helped to establish public health measures against COVID-19 [9–11]. This paper uses a media content analysis to examine reporting on international travel restrictions in Canada during the COVID-19 pandemic, how these restrictions aligned with the IHR 2005, and how the narrative around international travel evolved over the course of the pandemic.

## Methods

### Data collection

For this paper, we conducted a media analysis of Canada’s response to international travel during the COVID-19 pandemic. All articles published between January 1, 2020 and May 31, 2020 in Canada’s top three national newspapers by circulation (i.e., *The Globe and Mail, The National Post* and *The Toronto Star)* were screened for content. The Canadian Major Dailies - ProQuest database was used to identify newspaper articles that fit these criteria. This database provides access to more than 20 of Canada’s top national and regional newspapers in full-text format [15]. It also provides full-text versions of other document types including news articles, commentaries, correspondence, editorials, essays, letter to the editor, speeches, and government documents.

### Inclusion criteria

Four types of newspaper text formats were included in our analysis – news articles, editorials, commentaries and interviews. The federal travel rules and regulations reported in the newspapers were triangulated with the publications of federal departments and agencies such as Global Affairs Canada, Transport Canada, the Public Health Agency of Canada (PHAC), and Canada Border Services Agency (CBSA). We included content published between January 1, 2020 and May 31, 2020 using the key search term “COVID-19 AND International Travel”. Multiple keywords were initially used to search for articles, including “coronavirus AND travel”, “coronavirus AND international travel”, and “COVID-19 AND travel AND coronavirus” giving us between 2000 to 3000 search results each. To keep the search contained, we used “COVID-19 AND International Travel” as our only search terms. Only articles published in English were included.

### Data preparation and analysis

We developed a data extraction form to document data obtained from the articles. Two reviewers manually extracted content independently and in duplicate. We conducted a qualitative media content analysis [16] using an inductive coding approach wherein emergent themes were identified and extracted from the articles [17–20]. The analysis focused on federal and provincial governments’ guidelines and policies on international travel and changes in their position over time. The tone of the media articles (i.e., positive, neutral, or critical) was also identified based on our qualitative assessment of each article’s narrative. We also indicated whether the articles made reference to scientific and/or expert advice regarding international travel. Due to the evolving nature of the discourse around international travel, the extraction form and coding framework were modified as needed to capture emergent themes through a collaborative and iterative process. Two reviewers (KSR and SSM) were involved in the document identification, review, data extraction, and analysis. The titles and full text of the articles were screened independently by the two reviewers. Any disagreements in full-text selection and data extraction were resolved by consensus. To reach intersubjectivity in qualitative coding, the two reviewers independently developed code lists and merged them into one final coding list with common codes for data analysis. The inter-rater reliability for document screening and selection was tested using Cohen’s kappa co-efficient (κ) scale.

## Results

**Fig. 1:**
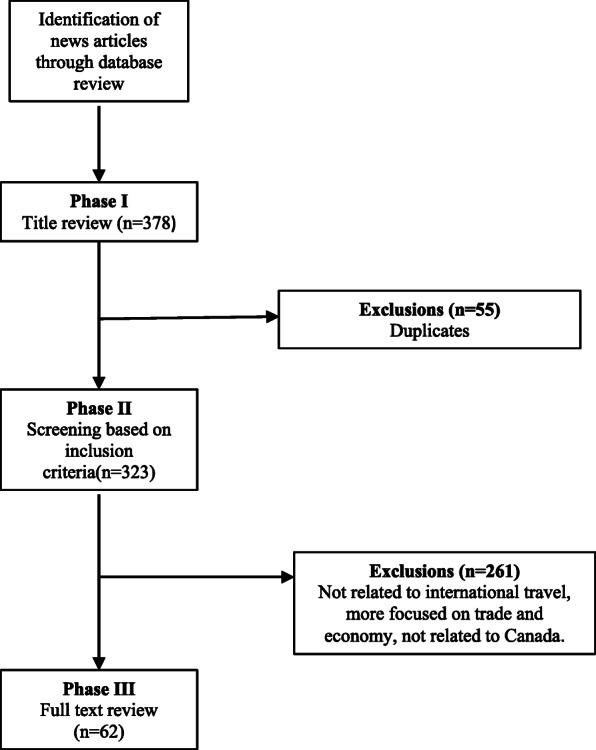
Flow chart for screening process

Our search yielded a total of 378 articles across the three newspapers. After removing duplicates (*n* = 55), the remaining 323 articles were screened based on our inclusion criteria. A final total of 62 articles were included for analysis. Reasons for exclusion are shown in Fig. 1. Inter-rater reliability testing revealed strong agreement between the two independent reviewers (κ = 0.830).

**Fig. 2:**
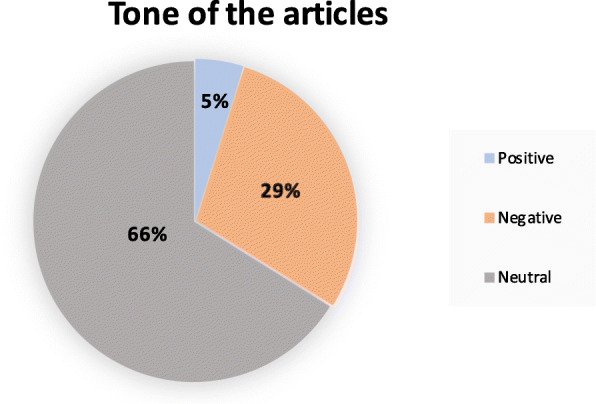
Overall media tone

The highest number of articles came from *The Globe and Mail* (*n* = 24, 39%), followed by *The Toronto Star* (*n* = 19, 31%) and *The National Post* (*n* = 17, 27%). The majority of articles struck a neutral tone (*n* = 41, 66%) (Fig. 2)

**Fig. 3:**
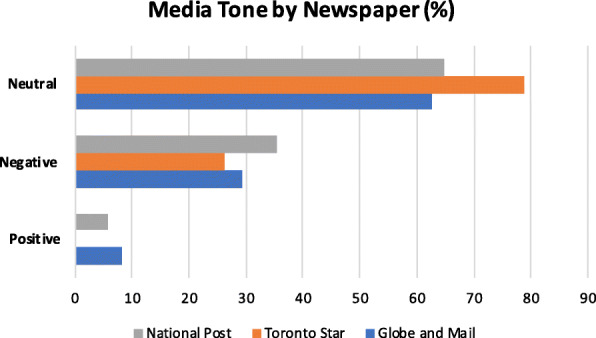
Media tone by newspaper

The tone within each newspaper was generally neutral, but the largest proportion of negative articles appeared in *the National Post*, where 6 of 17 articles were negative (35%). A detailed breakdown of the tone within each newspaper is presented in Fig. 3.

### Thematic analysis

Three major themes were identified within the articles. These included: 1) The role of scientific and expert evidence in implementing travel restrictions; 2) Federal legislation, regulation and enforcement of international travel measures; and 3) Compliance with WHO guidelines in travel restriction policy- and decision-making.

#### The role of scientific and expert evidence in implementing travel restrictions

Despite measures taken to restrict international travel as early as January, the media criticized the Canadian response as inadequate and slow. The federal government justified their decisions not to implement harsher border restrictions by citing scientific evidence:

“*[W]e are not closing the border to any further steps, but we will make those decisions based on what science tells us” – Prime Minister Justin Trudeau* [21].

“*I think Canadians think that we can stop this at the border, but what we see is a global pandemic meaning that border measures actually are highly ineffective and in some cases can create harm” – Health Minister Patty Hajdu* [21].

Similarly, articles cited Taiwan, South Korea, and Singapore as examples of countries that had demonstrated adequate preparedness and response; these were regions that quickly implemented widespread thermal testing and travel restrictions [22, 23]. By contrast, the media reported that the Canadian government deemed thermal testing of returning travellers unnecessary, citing a lack of scientific evidence in favour of the practice. Specifically, during the Severe Acute Respiratory Syndrome (SARS) outbreak in 2003, 2.3 million travellers were screened using thermal tests at Canadian airports, but these tests were later found to be ineffective in detecting SARS cases [24, 25]. Until March 10, the federal government continued to assert to the House of Commons Health Committee that the pandemic presented a low risk to Canadians [26]. It was not until March 18 that Canada closed its borders to most international travellers [27].

Despite the federal government’s stance, articles described actions implemented at the provincial level that did not align with federal action, including mass testing, screening of international travellers, stricter enforcement of quarantine orders, and recommendations for the use of masks [24, 26, 28–30]. Other articles reported that the provincial leaders of Alberta, New Brunswick and Prince Edward Island saw value in the travel restrictions implemented by other countries, including closing international borders [28, 31].

#### Media scrutiny of federal legislation, regulation and enforcement of international travel measures

On March 25, 2020, the federal government announced an emergency order under the *Quarantine Act, 2005,* which permits the screening of travellers entering Canada. Under the emergency order, any person entering Canada was required to self-isolate for 14 days regardless of whether or not they had symptoms of COVID-19 [32]. However, the media reported that provincial and federal travel screening measures and self-isolation guidelines were difficult to understand and inconsistent across jurisdictions [28, 30]. In particular, the media noted that self-isolation for healthcare workers varied by province and over time [30]. Restrictions were also implemented for travel between provinces, raising concerns over “mobility rights” outlined in the Canadian Charter of Rights and Freedoms. However, the Supreme Court of Newfoundland and Labrador upheld the provincial order barring an individual from entering the province and emphasised that the province’s travel restrictions were consistent with the Constitution in the interest of public health and contained the pandemic’s spread [33]. By early April, at least eight provinces and territories had some sort of border check points in place, but the content of these restrictions varied by jurisdiction (Table 1).

**Table 1:** Entry and exit control for inter-provincial movement

Province name	Travel restrictions or measures
**Quebec**	On April 1, 2020, the Quebec government effectively divided Canada’s Capital Region by placing checkpoints on the border between Ottawa, Ontario and Gatineau, Quebec. To block all non-essential travel into the province, the Quebec government also set up checkpoints on major roads leading to more remote areas of Quebec, as well as at the Quebec-U.S. border.
**Ontario**	No inter-provincial travel restrictions were placed.
**Manitoba**	Established checkpoints at main highways and airports to provide guidance about COVID-19 to travelers. Also issued travel advisories for domestic travellers entering the province to self-isolate for 14 days.
**Saskatchewan**	Although no domestic travel restrictions were placed, the government recommended that people self-monitor for symptoms if they have traveled outside of Saskatchewan, but within Canada.
**Alberta**	No inter-provincial domestic travel restrictions were placed.
**British Columbia**	No inter-provincial domestic travel restrictions were placed.
**Yukon**	All travelers entering the territory must self-quarantine for 14 days. This includes anyone returning home from other provinces and territories by road or air, as well as Yukoners returning home by road from Alaska.
**North West Territories**	All travellers into the territory by non-residents were prohibited with a few exceptions (such as those transporting essential goods and essential service workers).
**Nunavut**	Issued travel ban that restricts all entry into the territory aside from some specific exceptions like residents and essential workers. Furthermore, all residents returning must self-quarantine before entering Nunavut.
**Prince Edward Island**	Anyone coming into Prince Edward Island must self-isolate for 14 days following all out of province travel, including within Canada and the USA. Exceptions include essential service workers and flight crews.
**Newfoundland and Labrador**	All travellers entering the province must self-isolate for 14 days.
**Nova Scotia**	Anyone entering Nova Scotia must self-isolate for 14 days.
**New Brunswick**	As of March 15, 2020, anyone entering the province (aside from some exceptions like essential workers) must self-isolate for 14 days.

Further federal regulation was enacted on April 14, 2020 when regulatory amendments under the Contraventions Act came into force. These changes provided increased flexibility for law enforcement agencies to issue fines to individuals in violation of the Quarantine Act [34]. These actions received mixed coverage from the media, with some articles calling for the use of the Emergencies Act to restrict movement within the country as well as upon return from international travel [35]. Others, however, argued that such sweeping measures would restrict the liberty of those infected with COVID-19 [36].

Some articles also criticized the federal government for adopting a preferential approach to the United States (US) when closing the borders to other countries [37]. Despite the fact that provincial data from Ontario, Quebec, Alberta and British Columbia showed that many of Canada’s early COVID-19 cases came from the US [37], the federal government was slower to implement travel restrictions against the US compared to the action taken against other countries.

When it came to enforcing travel measures, the role of the CBSA – the federal law enforcement agency responsible for border control, immigration, and customs services in Canada – was closely scrutinized by the media. Despite the CBSA reporting that its officers were well-trained to identify visible signs of illness and to ask screening questions about possible symptoms, CBSA was criticized for inadequate screening measures and a lack of transparency regarding screening for travel history and passenger nationality [38]. The media also reported that there were inadequate screening measures in place at Canadian airports and that many health officials were screening passengers for COVID-19 symptoms over the phone, potentially increasing the risk of dishonesty from travellers. The media reported that this practice was attributed to a lack of sufficient personnel to accommodate in-person screening of all passengers [25].

#### Compliance with WHO guidelines in travel restriction policy- and decision-making

In the early days of the pandemic, much of the media attention in Canada focused on the WHO’s handling of the pandemic [39, 40]. In accordance with the IHR 2005, the WHO recommended against travel or trade restrictions in response to COVID-19. The media reported the federal government’s concerns about the risk of travel restrictions increasing protectionist sentiment among the Canadian population [41], as well as the government’s confidence in and commitment to WHO guidance, with the Chief Public Health Officer noting that “*we are a signatory to the International Health Regulations and we’ll be called to account if we do anything different”* [36]*.* This rationale was used to explain a reluctance to close national borders. This stance was often in conflict with the perspectives of provincial premiers, some of whom argued that the federal government’s delays were contributing to the spread of COVID-19 [37] and resulting in individual provinces implementing their own travel restrictions.

## Discussion

Our media analysis indicates that, despite having a comprehensive plan for meeting the IHR requirements regarding PHEICs at points of entry [42], Canada’s implementation of international travel restrictions in the early days of the COVID-19 pandemic was viewed by the media as inadequate. The federal government relied strongly on WHO’s advice and on scientific evidence for border closure and travel restrictions, despite a previous recommendation issued by the 2003 SARS commission that “*reasonable steps to reduce risk should not await scientific certainty*” [39, 43].

Specific media criticisms suggested that earlier action by the federal government may have reduced the number of COVID-19 cases imported into Canada [37]. Furthermore, the consolidation of provincial data on the sources of new cases may have offered a window of opportunity for the federal government to develop a coordinated response with provincial governments to contain the early spread of COVID-19 in the country. Instead, the federal government’s inaction was viewed as prompting provincial governments to develop their own international and inter-provincial travel restrictions. While critical of federal government’s travel restrictions for international travellers, the media also reported Canada’s distinction as one of the few countries that exempted maritime crews from travel restrictions to facilitate international trade [44].

The media play an essential role in the science and policy interface by communicating scientific information to the public and policymakers [45]. One policymaking model specifically describes the media’s role in the policy process as that of a key disseminator of scientific information for health emergencies [46]. Like the earlier pandemics of SARS (2003), H1N1 (2009), and MERS (2012), the international and Canadian media significantly contributed to the COVID-19 infodemic (i.e., an epidemic of rapidly spreading and far-reaching information about the pandemic) [47–49].

Our media analysis of three Canadian newspapers suggests that in the early days of the pandemic, much of the media attention remain focused on restricting the international travel and quarantine measures for international travellers. Media studies conducted elsewhere reported similar findings [9, 11, 47]. By contrast, a previous analysis by Irwin regarding media coverage of Sweden’s pandemic response suggested that the international media failed to communicate the complexities of science and policy in and instead presented multiple narratives. These narratives included: normalcy of life, herd immunity strategy, expert advice not being considered, non-compliance with WHO recommendations, failing to contain the pandemic within the country, and Swedes’ trust in the government. Irwin argues the importance of fact-checking and source critique and the need for precision when presenting data and statistics while evaluating pandemic policies [11]. Conversely, the Australian media remained objective in reporting the pandemic and government’s response [9] by framing the narrative specifically around “action” and “consequence” [9, 50], an approach that was effective for earlier outbreaks of Mad Cow Disease, West Nile virus, and Avian Flu [50].

Our media analysis of three newspapers did not find the same narrative framing used in the case of the Australian media but found some similarities to the narratives presented around Sweden’s pandemic response. Specifically, similarities were noted in Canada’s difficulties containing the pandemic, and Canadian trust in the federal government’s response, while differences were found in Canada’s consideration of expert advice and compliance with WHO’s recommendations for restrictions against international travel. In Canada, both the federal and provincial governments implemented unprecedented public health measures, including international travel restrictions. The studies conducted in Canada found that, through appropriate and best data visualisation strategies, public health experts and governments were able to successfully convince citizens that the public health measures being taken were necessary [51]. The media also reported Canada’s embrace of a rules-based international order - a shared commitment by all countries to conduct their activities by agreed international norms [52, 53] - by complying with the WHO’s advice and with the IHR in delaying international travel restrictions.

Multiple modelling studies conducted around the effectiveness of international travel restrictions for the influenza pandemic and other outbreaks determined that international travel restrictions were of limited benefit in slowing the global spread of pathogens [54–59]. More recent studies have shown similar evidence for the COVID-19 pandemic [60]. Notwithstanding scientific evidence, implementation of travel regulations appeared to be at least partly political. For example, in Zimbabwe, the government’s decision not to institute travel restrictions against Chinese citizens drew sharp criticism from civil society and the main political opposition party. In Canada, the federal government’s delayed implementation of international travel restrictions until mid-March received criticism from political parties, provincial premiers, civil society, and media alike [61].

The effectiveness of international travel restrictions such as border closures is not always definitive, but evidence suggests that that the shutdown of international airports and border closures prevented spillover across countries in the early phase of the pandemic [62, 63] while countries that delayed closing their border may have had higher infection rates [64]. Given these potential benefits, there is a need to review and potentially revise current IHR guidelines regarding travel restrictions during PHEICs [63, 65, 66].

### Limitations

This study is limited to news articles published between January 1st and May 31st, 2020. The majority of articles captured were published in March and April. The data were limited to newspaper articles from three newspapers and may not be representative of Canadian media as a whole. As only newspapers were used from the database and the article formats were limited to news articles, interviews, editorials, and commentaries, excluding speeches, essays, letters to the editor, government documents, we may have missed information outside of our inclusion criteria. Moreover, the term “coronavirus” was more commonly used than “COVID-19” in those months and non-inclusion of this search term may have eliminated some key information. Furthermore, restricting articles to those published in English language may also serve as a limitation.

## Conclusion

As early as January, Canada implemented border screening measures for international travellers, before closing the borders in March. However, our media analysis of Canada’s international travel measures for the COVID-19 pandemic suggests that these measures were viewed as inadequate and occurred too late. By the time Canada imposed travel restrictions in late March, more than 4 million people had already entered the country. Media reporting suggested that the time for Ottawa to act broadly and in the national interest was in January or February, when strong actions in areas of clear federal responsibility could have had greater impact. The media narrative about the pandemic public health measures that were taken should be considered in discussions of national pandemic preparedness and response, as the pandemic is far from over in Canada and elsewhere.

## Data Availability

The data that support the findings of this study are available from the three Canadian newspapers ((i.e., *The Globe and Mail, The National Post and The Toronto Star)* and available in the public domain.
